# Intratendinous injections of platelet-rich plasma: feasibility and effect on tendon morphology and mechanics

**DOI:** 10.1186/s40634-014-0018-5

**Published:** 2015-03-01

**Authors:** John J Wilson, Kenneth S Lee, Connie Chamberlain, Ryan DeWall, Geoffrey S Baer, Marcus Greatens, Nicole Kamps

**Affiliations:** Division of Sports Medicine, Department of Orthopedics and Rehabilitation, University of Wisconsin School of Medicine and Public Health, 1685 Highland Avenue, Madison, Wisconsin 53705 USA; Department of Biomedical Engineering, University of Wisconsin School of Medicine and Public Health, Madison, Wisconsin 53705 USA; Department of Radiology, University of Wisconsin School of Medicine and Public Health, Madison, Wisconsin 53705 USA

**Keywords:** Platelet-rich plasma, Intratendinous injections, Tendon mechanics

## Abstract

**Background:**

Intratendinous injections may have important effects on the properties of collagen microarchitecture, morphology, and subsequent mechanical properties of the injected tendon. The purpose of this study was to examine the effects of intratendinous PRP injections; the injectant retention within tendons, the distribution of intratendinous injectant, and whether intratendinous injection or needle fenestration alters tendon morphology or mechanics.

**Methods:**

Design: Controlled Laboratory Study.

Interventions: In the first part of the study, 18 lamb extensor tendons were selected to receive methylene blue-containing PRP injection (PRP/MB), methylene blue only injection (MB), or needle fenestration. The volume of retained injectant was measured and injectant distribution and tendon morphology were examined microscopically. In the second portion of the study, 18 porcine flexor tendons were divided into control, needle fenestration, or saline injection groups. Young’s Modulus was then determined for each tendon under 0-4% strain.

Main outcome measures: 1) Injectant volume retained; 2) Injectant distribution; 3) Post-injection/fenestration alterations in morphology, biomechanics.

**Results:**

Intratendinous injectant is retained within the tendon. The difference between PRP and PRP/MB groups was not significant (p = 0.78). Intratendinous spread of the injectant solution within the tendon occurs primarily in the proximodistal direction, with very little cross-sectional penetration. Intratendinous injections resulted in microscopic morphology disruption (e.g., separation and disorganization of both the collagen bundles and cellular distribution).

There were significant differences in Young’s Modulus between control (E_ctrl_ = 2415.48) and injected tendons (E_inj_ = 1753.45) at 4% strain (p = 0.01). There were no differences in Young’s Modulus between fenestrated and control tendons.

**Conclusions:**

Intratendinous PRP injections are retained within the tendon, and primarily distributes longitudinally with minimal cross-sectional spread. Intratendinous injections may alter tendon morphology and mechanics.

## Background

Tendinopathy is a common source of pain and functional impairment, accounting for 30-50% of sports-related overuse injuries and affecting large numbers of laborers who perform repetitive movements (Scott and Ashe 2006; Forde et al. 2005). Most tendinopathies develop gradually as a result of repeated physical strain (Kazemi et al. 2010; Kraushaar and Nirschl 1999). The strenuous and repetitive nature of recreational and occupational activities can lead to degenerative tendon alterations, characterized by collagen fiber disorganization, neovascularity, tendon thickening, increased cellularity, matrix disorganization, tenocyte apoptosis, and the clinical manifestations of pain and dysfunction (Sharma and Maffulli 2006; Jozsa and Kannus 1997; Cook and Purdam 2009). There is generally a paucity of inflammatory cells (Khan et al. 1999). Tendinopathies are often refractory to conservative treatment modalities (e.g. rest, non-steroidal anti-inflammatory drugs, physical therapy, bracing, corticosteroids) and such treatments have not been proven to affect healing at the cellular level (Mishra and Pavelko 2006).

Platelet-rich plasma (PRP) injection therapy has shown promise as an effective therapy for chronic tendinopathy (Mishra and Pavelko 2006; Filardo et al. 2010; Barrett 2004; Thanasas et al. 2011; Peerbooms et al. 2010; Gosens et al. 2011; Mishra et al. 2014). Platelets, which are small fragments of megakaryocytes that are essential for normal coagulation, also play a vital role in tissue healing via their release of platelet-derived growth and differentiation factors at the site of injury (Lee et al. 2011). PRP injections are proposed to stimulate and augment regenerative healing of degenerative tissue through the coordinated action of the PRP growth factors and cytokines (Sampson et al. 2008; Anitua et al. 2006; Anitua et al. 2005; Molloy et al. 2003). After intratendinous injection, platelets are activated upon exposure to collagen and local release of thrombin induced by needle fenestration. Upon platelet activation, platelet-derived growth factors (PDGFs) are released locally where they exert various responses through local cellular and cytokine signaling pathways necessary for cell proliferation and remodeling at the cellular level (Everts et al. 2006).

Recent studies have demonstrated beneficial results of PRP therapy in chronic tendinopathies, including those refractory to conventional treatment modalities (Mishra and Pavelko 2006; Filardo et al. 2010; Barrett 2004; Thanasas et al. 2011; Peerbooms et al. 2010; Gosens et al. 2011; Mishra et al. 2014). Other research has demonstrated beneficial effects of saline injections or simple percutaneous tendon fenestration for tendinopathy (Housner et al. 2009; McShane et al. 2006; McShane et al. 2008; Testa et al. 2002; Testa et al. 1999; Chan et al. 2008; Crisp et al. 2008). Despite the emerging evidence for the efficacy of PRP therapy, it is not known whether the beneficial effects of PRP injection therapy are a result of the PRP and its inherent growth factors, or simply due to the controlled tendon trauma and localized bleeding caused by the tendon fenestration. Therefore, it is important to understand whether it is technically feasible for an injectant to remain within the tendon after intratendinous injection in order for the hypothesized clinical benefits of the injectant (e.g., PRP and PDGFs) to be realized.

Similarly, little is known regarding the effects intratendinous injections may have on the properties of collagen microarchitecture, morphology, and subsequent mechanical properties of the injected tendon. Tendons do not act as rigid structures, but rather demonstrate viscoelastic properties that allow them to stretch, store, and release energy transmitted from their associated muscle group (Maganaris 2002; Alexander 1988; Zajac 1989). Several studies have demonstrated that tendinopathy alters these mechanical properties in affected tendons, resulting in increased cross-sectional area, less tendon stiffness, and lower Young’s Modulus (Helland et al. 2013; Arya and Kulig 2010). The mechanical alterations due to tendinopathy result in an inherently weakened tendon structure. Intratendinous injections or fenestration may have additional important short-term and long-term effects on the mechanical properties of tendons. Understanding these consequences is necessary for optimizing the tendinopathy treatments while minimizing complications.

The goals of this study are to determine the effects of intratendinous PRP injections including, (1) the retention of injectant within the tendon, (2) the distribution of PRP after intratendinous injection, (3) whether intratendinous injection or tendon needle fenestration alters tissue morphology, and (4) whether intratendinous injection or tendon needle fenestration alters tendon mechanical properties.

## Methods

### Part one

Eighteen fresh frozen lamb extensor carpi radialis tendons obtained from a local abattoir were dissected, weighed, and then divided into three subgroups by a single researcher (MG). PRP was first diluted with .014% methylene blue. Six tendons were selected to each receive a single 0.25 mL injection of methylene blue-containing platelet-rich plasma (PRP-MB), six were prepared to each receive a single 0.25 mL injection of methylene blue alone (MB), and six served as controls and received only a needlestick without injection. Platelet-rich plasma was obtained from 20 mL of whole blood, drawn from the investigator (MG) in lab. The whole blood was spun in a benchtop centrifuge (Sorvall Legend, RT+, Thermo Fisher Scientific, Waltham, MA) for 10 minutes at 312 G, yielding 3.5 mL of un-activated (e.g., using thrombin or calcium chloride) PRP. The injections were administered using a 22-gauge, 1.5” needle (Magellan®). The needle was inserted longitudinally at a 45-degree angle at the junction of the proximal 1/3 and middle 1/3 of each dissected tendon. Immediately following injection or needlestick, each tendon surface was gently dabbed dry to remove any extratendinous fluid. The wet weights of the tendons were obtained before and immediately after injections using a light balance (Mettler Toledo, Columbus, OH) with a repeatability of 0.0001 g. The difference between pre- and post- injection weights was calculated to determine the volume of injectant remaining within the tendon. The tendons were then trimmed to fit into 4.5 cm cassettes, embedded in optimal cutting temperature (OCT) medium and flash frozen in liquid nitrogen. Longitudinal sections of the frozen samples were cut at 10 μm thickness, mounted on Superfrost Plus (Thermo Fisher Scientific, Waltham, MA) microscope slides and used for H&E staining. Representative tendon micrographs of each tendon were collected using a camera-assisted microscope (Nikon Eclipse microscope, model E6000, Nikon Instruments, Inc, Mellville, NY with an Olympus camera, model DP79, Olympus Imaging America, Inc, Center Valley, PA).

The distribution of the injectant in each sample was then measured longitudinally (maximum proximodistal spread) and transversely (maximum diameter of injectant spread within the tendon) using the H&E micrographs. The extent of collagen fiber microarchitecture disruption was also qualitatively assessed.

Tendons embedded in cryostat gel were then sectioned into 10 μm slices, and mounted onto slides for further analysis. An experienced tissue histologist (C.C.) then evaluated injected tendons to measure longitudinal (maximum proximodistal spread) and cross-sectional (maximum diameter of injectant spread within the tendon) distribution of the injectant. The same histologist also qualitatively assessed tendon collagen fiber microarchitecture disruption.

### Part two

**Figure 1:**
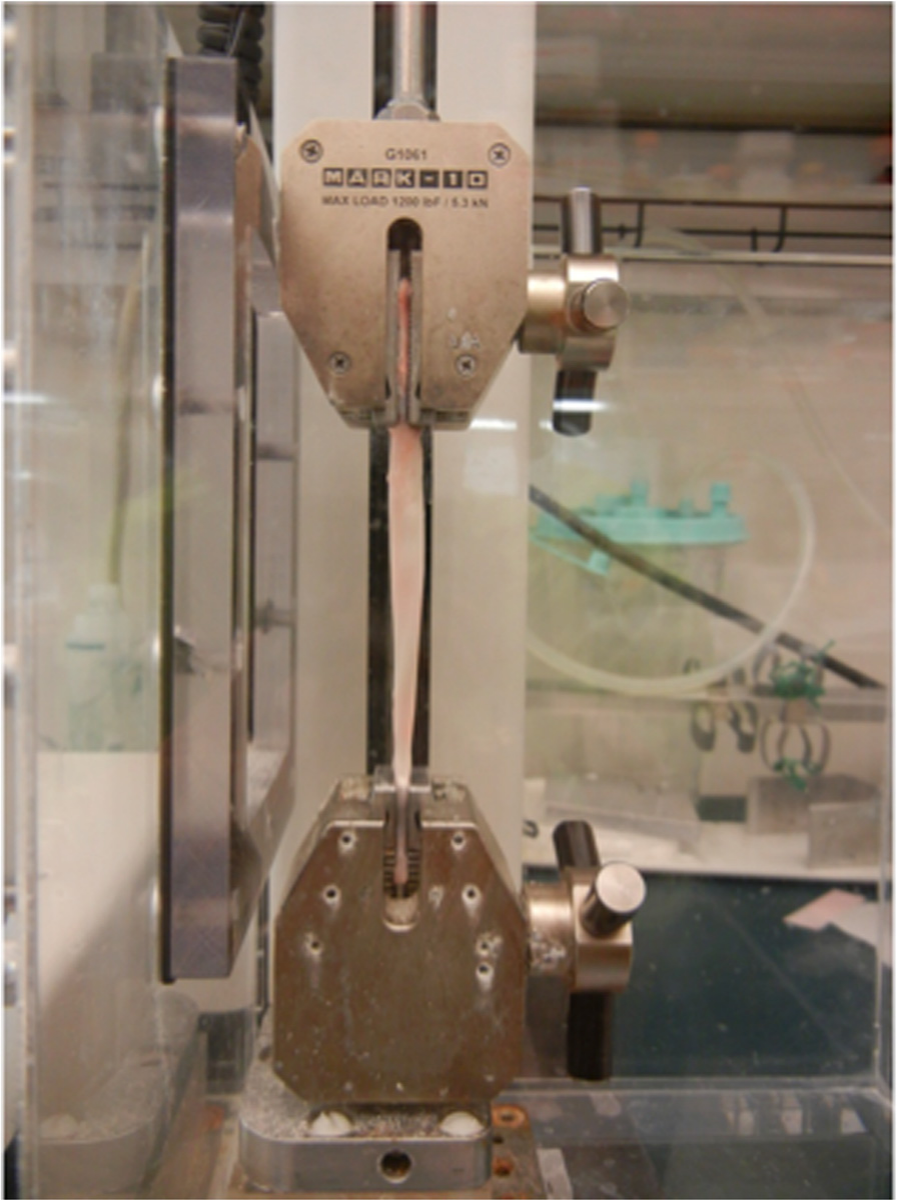
**Mark-10 force measurement system.**

Eighteen similarly sized porcine flexor digitorum tendons (3^rd^ digit) were excised by a single researcher (NK) from 18 forelimbs obtained from a local abattoir. Specimens were kept hydrated in physiologic buffered saline until loading into the mechanical test system. Porcine flexor tendons, rather than lamb extensor carpi radialis tendons, were utilized for mechanical testing, since they were comparable in size (length, weight, circumference) to the lamb extensor tendons sectioned in part one, and were conveniently available for testing. Six tendons served as controls, six received a single needle fenestration, and six were selected to receive a single 0.25 mL bolus injection of 0.9% saline within the tendon. The injections and fenestrations were performed using a technique identical to part one. Immediately following the tendon treatments, a Mark-10 ESM301 Test Stand (Mark-10 Corp; Copiague, NY) was used to stretch the tendons from 0-4% strain (Figure 1). Force was measured with a MARK-10 Series 5 Force Gauge (Mark-10 Corp; Copiague, NY), and the load and displacement data were recorded with MESUR^TM^ gauge load and travel analysis software (Mark-10 Corp; Copiague, NY). From these data stress (Force/Area) and strain ((l-l_0_)/l_0_) were calculated. The Young’s Modulus was calculated as (stress/strain). The measurements obtained for each tendon within each group were then averaged for comparative analysis and paired t-tests were used to examine the effect of increased strain on Young’s Modulus (*E*) for each treatment group.

## Results

1. Intratendinous retention of injectant
  Pre-injection tendon weight did not significantly differ among the three groups as determined by one-way ANOVA (F(2,15) = 0.75, p = 0.49). The post-injection weight of the tendons in the PRP-MB group increased by an average of 0.22 ± 0.03 g (Table 1). Based on the measured density of this fluid (1.40 g/mL), this correlates to an average of 62.7% of the 0.25 mL injectant retained per tendon in this group. The post-injection weights of the MB tendons increased by an average of 0.23 ± 0.10 g, correlating to 52.4% injectant volume retained, again based on the measured density of the injected fluid (1.76 g/mL). The average retention of MB versus PRP-MB was not significantly different (p = 0.78). As expected, the control needle fenestration tendons did not have a significant change in weight.

**Table 1:** **Average volume of injectant retained within tendons of MB only vs. PRP/MB groups**

**Group**	**Avg. pre-weight (g)**	**Avg. post-weight (g)**	**Avg. increase in weight (g)**	**% Injectant retained**	**P-value**
**MB Only**	2.78 ± 0.26	3.00 ± 0.26	0.23 ± 0.04	54.4	0.78
**PRP/MB**	2.39 ± 0.21	2.39 ± 0.21	0.22 ± 0.01	62.7	

Data are expressed as the mean ± S.E.M.
2. Distribution of injectant
  The mean proximodistal penetration of injectant for the PRP-MB group was 2.91 ± 0.32 cm (Table 2). This was not significantly different from the MB group, which had a mean proximodistal penetration of 2.77 ± 0.40 cm (p = 0.80). The PRP-MB group had a mean cross-sectional penetration of 0.21 ± 0.06 cm, significantly less than the average 0.27 ± 0.04 cm seen in the MB group (p = 0.02).

**Table 2:** **Average proximodistal and cross-sectional distribution of injectant in PRP/MB vs. MB only groups**

**Proximodistal**	**Avg. spread (cm)**	**P-value**
MB only	2.77 ± 0.40	0.80
PRP/MB	2.91 ± 0.32	
**Cross-sectional**		
MB only	0.27 ± 0.04	0.02
PRP/MB	0.21 ± 0.06
3. Tissue morphology following injection
  Each tendon was examined microscopically after injection or fenestration for evidence of normal tendon morphology disruption. Injected tendons (MB and PRP-MB) consistently demonstrated disruption of normal tendon morphology compared to control tendons (needlestick). Characteristic findings of injected tendons included separation of adjacent collagen bundles and disorganization of both the collagen bundles and cellular distribution (Figure 2).

**Figure 2:**
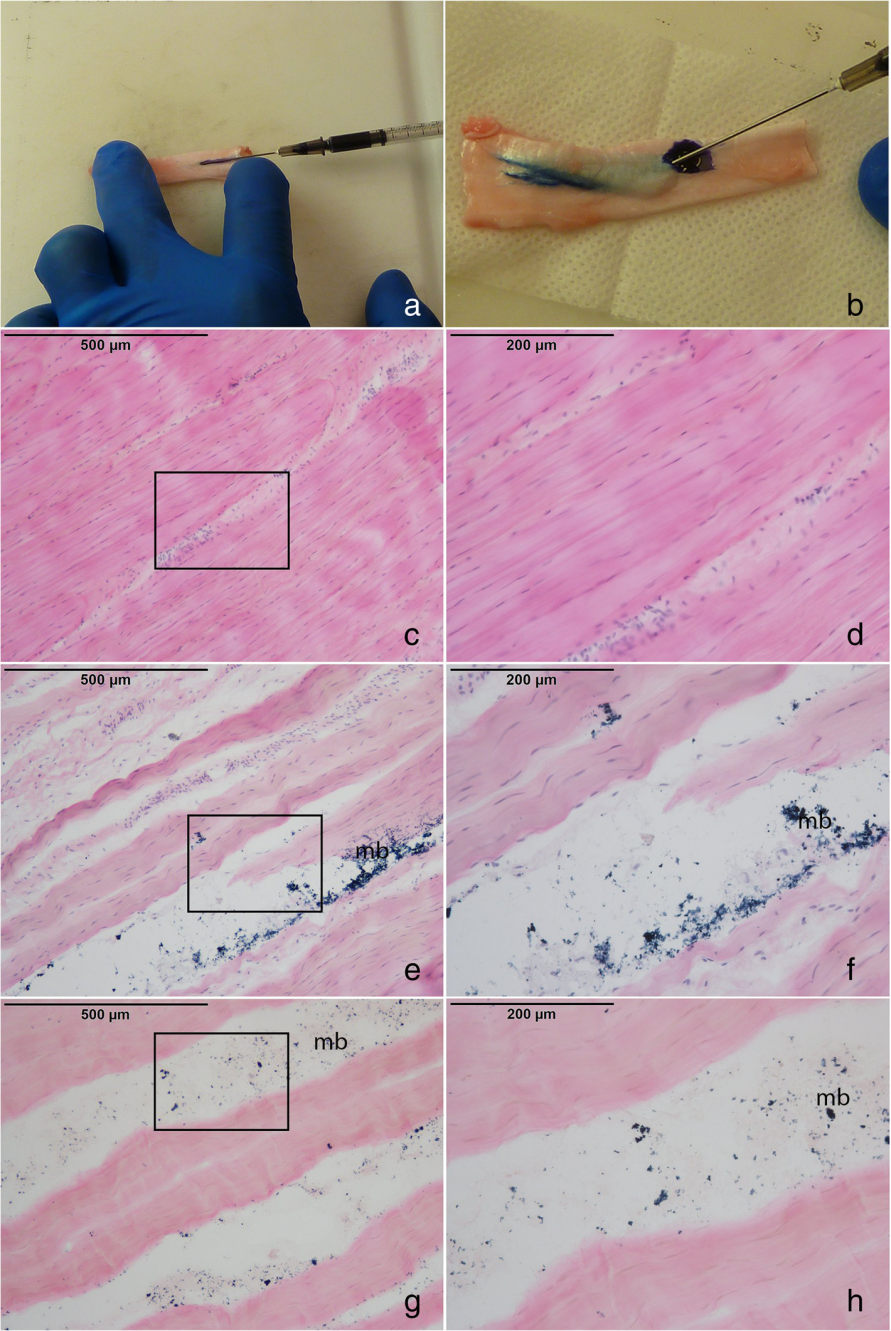
**Morphologic changes in injected tendons versus control.** Figures 2 **a)** and 2 **b)** represent the injection process of the PRP/MB and MB into the lamb tendon. The tendon significantly expands as the solution is injected **(2b)**. H&E images of the **2c)** and **2d)** fenestrated tendon, **2e)** and **2f)** MB-injected tendon and 2 g) and 2 h) PRP/MB-injected tendon. Note the methylene blue staining (mb) within the open spaces between the collagen fibers. Rectangles in **2c)**, **2e)**, and **2 g)** represent the area captured in images **2d)**, **2f)**, and **2 h)**.
4. Effect of intratendinous injection and fenestration on tendon mechanical properties
  Young’s modulus (E), the slope of the stress–strain curve, was used as a measure of the tissue mechanical property in the context of multiple different levels of tensile strain (0-4%) upon the tissue (Table 3). The injection group did show a lower average Young’s Modulus (E = 1753.45 kPa) at 4% strain compared to control tendons (E = 2415.48 kPa), demonstrating that injected tendons were significantly less stiff than controls under the highest level of strain tested (p = 0.01).

**Table 3:** **Young’s modulus of injected vs. control tendons at various levels of strain**

	**1% Strain**	**2% Strain**	**3% Strain**	**4% Strain**
**Average E Control (kPa)**	53.68	444.81	1295.60	2415.48
**Average E Injection (kPa)**	63.43	373.55	893.59	1753.45
**P-value**	0.66	0.56	0.09	0.01
  The effect of needle fenestration on the mechanical properties of tendons was then analyzed, again measured as Young’s Modulus (E) (Table 4). There was no significant difference in Young’s Modulus at any level of strain in the fenestrated group compared to the control group.

**Table 4:** **Young’s modulus of fenestrated vs. control tendons at various levels of strain**

	**1% Strain**	**2% Strain**	**3% Strain**	**4% Strain**
**Average E Control (kPa)**	53.68	444.81	1295.60	2415.48
**Average E Fenestration (kPa)**	60.03	532.03	1333.34	2358.95
**P-value**	0.82	0.51	0.87	0.76

## Discussion

Intratendinous injections of PRP for tendinopathy are thought to rely on the intrinsic properties and interplay between the growth factors contained within plasma and the platelet alpha granules (Lee et al. 2011). Some of the important growth factors contained within PRP include platelet-derived endothelial growth factor, transforming growth factor-beta, vascular endothelial growth factor, fibroblast growth factor, epidermal growth factor, and insulin-like growth factor-1 (Anitua et al. 2004; Lee et al. 2011). Several *in vitro* and *in vivo* studies have demonstrated the ability of PRP to improve healing at the cellular level (Schnabel et al. 2007; Majewski et al. 2009; Zhang and Wang 2010; de Mos et al. 2008; Kajikawa et al. 2008; McCarrel and Fortier 2009). The rapidly expanding use of PRP clinically, however, has outpaced the scientific understanding of the precise mechanisms by which such treatments influence healing. If the hypothetical benefits of PRP stem from its ability to locally affect cellular healing, then a relevant amount of PRP injectant must remain in the tendon into which it was injected. It is also important to understand what effects, if any, these intratendinous injections have on the integrity and biomechanical properties of tissue. To date, there is limited understanding of the distribution of intratendinous injections, and, just as importantly, the resulting histological and biomechanical effects on tissue. Understanding the behavior of injected PRP and its effects on the tissue have important implications for optimizing patient care and outcomes.

We evaluated the intratendinous retention and distribution of PRP into a tendon using ex vivo laboratory techniques and post-injection histological tissue evaluation. We then examined the biomechanical tissue properties of fenestrated and injected tendons to determine whether these treatments have any immediate detrimental effects on tissue. Our results demonstrate that intratendinous PRP injections are not only feasible, but the majority of injectant remains within the tendon following injection. One study by Wiegerinck and colleagues looked at the distribution of PRP injected into cadaveric Achilles tendons. The results demonstrated a wide, poorly localized distribution of injectant upon post-injection dissection and evaluation of the tendon and inscribing tissues (Wiegerinck et al. 2011). The results of our study are similar in that, while the majority of injectant remained within the tendon, there was also a moderate amount of injectant that escaped the tendon substance, resulting in volume retention of less than two-thirds of the injectant in all injected tendons in this trial. A recent retrospective study of pre-and post-injection ultrasound imaging of tendons injected with PRP or autologous blood verified that injectant remained within the tendon of interest following injection, but injected solution was also visualized in tissues adjacent to the injected area (Loftus et al. 2012). Quantifying the volume of fluid remaining within the tendon immediately after the injection however, was not possible using the aforementioned study methods. Our study agrees with their findings, but quantifies the amount of solution remaining within a tendon after injection. Using histological evaluation, we were also able to quantitatively describe the characteristics of intratendinous spread of the fluid and the qualitative effects on tissue morphology immediately after injection. Loftus and colleagues suggest that the spread of injectant within a tendon proceeds more distally than expected, aligning with our findings of predominantly proximodistal spread. They did not specify, however, the degree of cross-sectional spread within the tissue, and hypothesized that peppering techniques may not be necessary given the non-local spread of injectant within the tissue (Loftus et al. 2012). Our results suggest that cross sectional spread is minimal, which may limit adequate distribution of injectant within tendon. This is consistent with the results demonstrated by van den Belt and colleagues in a similar in vitro study of intratendinous injections into horse flexor tendons (van den Belt et al. 1993). This observation was particularly evident in PRP-injected tendons in our study and the significant difference between PRP and methylene blue only injections limiting cross-sectional spread may be related to platelet activation and clotting upon exposure to tendon collagen, resulting in even further reduced cross-sectional spread across collagen bundles. This may suggest that larger areas or volumes of tendinopathy may require a peppering technique to ensure adequate distribution within the pathologic tissues being treated, in contrast to the conclusions of Loftus. Multiple fenestrations associated with a peppering technique may also provide theoretical benefit in creating local bleeding and exposure to thrombin, a known activator of platelets and stimulus to an acute healing response. The PRP utilized in this study was not activated using thrombin or calcium chloride, and may differ from clinical applications in which activation of PRP is performed.

The morphology of tendon microarchitecture is noticeably altered following intratendinous injection with a fluid bolus, whereas needle fenestration did not appear to have a significant effect on visualized tendon morphology. This may help to explain why intratendinous injections resulted in alterations of biomechanical properties, specifically decreased stiffness (E), with higher strain forces. There was no significant effect of needle fenestration on the biomechanical properties of tendons compared to controls. This demonstrates that injected tendons were significantly less stiff at higher levels of strain than control tendons and suggests that intratendinous injections may weaken the injected tendon, at least immediately following injections.

The use of intratendinous injections of PRP is emerging as a therapy for various chronic tendinopathies (Mishra and Pavelko 2006; Filardo et al. 2010; Barrett 2004; Thanasas et al. 2011; Peerbooms et al. 2010; Gosens et al. 2011; Mishra et al. 2014). While there is optimism surrounding PRP therapy, the feasibility and biomechanical effects of intratendinous injections on the treated tendon tissue is largely unknown and may have important implications for determining the optimal treatment strategy for such tendinopathies. In a letter to the editor by Knobloch, et al. (Knobloch et al. 2008) in response to a published study of intratendinous injections for Achilles tendinopathy (Maxwell et al. 2007), the authors raised concerns regarding the unknown effect of intratendinous level of collagen distribution on the potential adverse effects of intratendinous injections. Our study specifically examined the biomechanical effects, but other deleterious effects on tendon beyond physical disruption and biomechanical detrimental effects are also hypothesized (Abate et al. 2014). Such proposed effects include risk of pressure-induced tissue necrosis and focal infections, as well as focal tendon hypoxia and elevated lactate concentrations in tendons with tendinosis (Knobloch et al. 2008). These effects should be considered for future study as well.

Further study is warranted at a number of levels to increase our understanding of the effects of intratendinous injections. While this study demonstrates that a significant portion of a 0.25 mL injectant can be retained, it is likely to vary considerably with different injection volumes and operator dependent techniques. It is also not known whether the retention and distribution of intratendinous injections performed in vivo would be altered by factors such as blood flow and forces placed on the tendons during normal motion in the extremity (Wiegerinck et al. 2014). These factors may affect the injectant retention and distribution within the tendon. Although injected tendons in this study demonstrated visual disruption of normal morphology and immediately altered mechanical properties at higher levels of strain, it is not known what effect these alterations have on long-term function, morphology, and overall health of tendons in vivo.

A recognized limitation of this study is the fact that two different species and tendon types were analyzed for convenience reasons. Although the tendons were similarly sized, and from the forelimb of the animal, it is plausible that extensor and flexor tendons do not behave similarly in their response to intratendinous injections or tendon strain due to inherent differences in their structural and mechanical properties at baseline. Pollock and Shadwick’s study on the relationship between mechanical properties of various limb flexor and extensor tendons in 18 different quadrupedal mammals with different body mass did not significantly differ in their mechanical properties (Pollock and Shadwick 1994). Therefore, for the purposes of this study, tendon similarities were felt to be sufficient to allow for an initial investigation of the potential effects of intratendinous injections.

Given the relatively small sample size in both parts of this study and the fact that all measurements were collected at a single point in time immediately after treatment administration, future studies with larger sample sizes and the ability to measure these variables at multiple points in time would be very beneficial in better characterizing the effects of intratendinous injections and how best to counsel patients receiving this treatment.

Though this study demonstrated alteration of tendon mechanics in injected tendons at 4% strain, it remains unknown whether similar alteration would be seen in vivo or to what significance this type of alteration may have on likelihood of re-injuries or rupture, which obviously must be considered when determining timing for return to activity or sport. Another limitation of this study is the fact that the effect of intratendinous injections and fenestration on mechanical properties of the tendon was only tested to a maximum of 4% strain. In normal movement and activity, tendons typically experience strains of less than 4%, predominantly due to uncrimping of the collagen fibers until reaching roughly 2% strain and then due to actual stretch of the collagen fibers as strains increase beyond that (Kelc Robi et al. 2013). When a tendon is strained beyond 4%, microfailure of individual fibrils begins to occur and macrofailure (e.g., rupture) of the tendon typically occurs when it is stretched to greater than 8-10% its original length (Kelc Robi et al. 2013; Woo et al. 2000). In porcine tendons, irreversible damage generally occurs at or above 6.5% strain (Duenwald-Kuehl et al. 2012). Thus, while our study suggests that intratendinous injections have the potential to alter tendon mechanics at strains on the upper limit of those produced with normal activity, further research is necessary to investigate whether tendon mechanics are also altered at higher levels of strain and whether or not the point of rupture is affected following these treatments. Similarly, further research is necessary to determine whether or not the observed alteration of mechanics is reproducible in an in vivo model and whether the addition of PRP to the injectant results in alterations different than those seen with saline injections. Our results suggest early tendon loading at low strain is likely acceptable, but it may be prudent to exercise caution and avoidance of high strain loading of tendons treated with intratendinous injection in the immediate period following injection. It is not known how long the mechanical effects observed in this study may persist.

## Conclusions

Intratendinous PRP injections are retained within the tendon, and primarily distributes longitudinally with minimal cross-sectional spread. Intratendinous injections may alter tendon morphology and mechanics.
